# DPO multiplex PCR as an alternative to culture and susceptibility testing to detect *Helicobacter pylori *and its resistance to clarithromycin

**DOI:** 10.1186/1471-230X-11-112

**Published:** 2011-10-17

**Authors:** Philippe Lehours, Elodie Siffré, Francis Mégraud

**Affiliations:** 1 Université de Bordeaux, Centre National de Référence des Campylobacters et des Hélicobacters, 146 rue Léo Saignat, 33000 Bordeaux, France; 2 CHU de Bordeaux, Hôpital Pellegrin, Laboratoire de Bactériologie, Place Amélie Raba Léon, 33076 Bordeaux cedex, France; 3 INSERM U853, 33000 Bordeaux, France

## Abstract

**Background:**

Macrolide resistance in *Helicobacter pylori *is the major risk factor for treatment failure when using a proton pump inhibitor-clarithromycin containing therapy. Macrolide resistance is due to a few mutations on the 23S ribomosal subunit encoded by the 23S rRNA gene. The present study aimed at investigating the performance of the dual priming oligonucleotide (DPO)-PCR kit named Seeplex^® ^ClaR-*H. pylori *ACE detection designed to detect *H. pylori *and two types of point mutations causing clarithromycin resistance in *H. pylori*.

**Methods:**

The performance of Seeplex^® ^ClaR-*H. pylori *ACE detection was evaluated on 127 gastric biopsies in comparison to conventional bacterial culture followed by the determination of susceptibility to clarithromycin by E-test, as well as by an in-house real-time PCR using a fluorescence resonance energy transfer (FRET) technology.

**Results:**

Considering culture as the reference test, the sensitivity of DPO-PCR and real-time FRET-PCR was 97.7% and 100% while specificity was 83.1% and 80.7%, respectively. However, both PCR were concordant in detecting 14 *H. pylori *positive cases which were negative by culture. Globally, E-test and DPO-PCR were concordant with regard to clarithromycin susceptibility in 95.3% of the cases (41/43), while real-time FRET-PCR and DPO-PCR were concordant in 95% (57/60).

**Conclusion:**

The DPO-PCR is an interesting tool to detect *H. pylori *on gastric biopsies and to study its susceptibility to clarithromycin in laboratories that cannot perform real-time PCR assays.

## Background

Macrolide resistance in *Helicobacter pylori *is the major risk factor for treatment failure when using a proton pump inhibitor (PPI)-clarithromycin containing therapy [1]. Macrolide resistance is due to a few mutations on the 23S ribomosal subunit encoded by the 23S rRNA gene [2,3]. These mutations (A2142C, A2142G, A2143G), are easy to detect by numerous molecular methods directly on gastric biopsy specimens and even on stool samples [4-7].

A new PCR format named DPO-PCR for "Dual Priming Oligonucleotide" was recently developed [8]. DPO-PCR is a multiplex PCR assay that increases specificity and sensitivity of detection compared to conventional PCR, by blocking non-specific binding sites therefore eliminating imperfect primer annealing. This new technology can be used for many applications in the field of *in vitro *diagnostics: simultaneous detection of multiple pathogens and of polymorphisms (SNPs), as well as simultaneous genotyping of multiple pathogen subtypes. DPO-PCR is based on a multiplex PCR using a DPO patented technology [8]. The structure of the DPO primers is fundamentally different from that of conventional primers. Indeed, the primer is divided into two parts by a 5 polydeoxyinosine linker which allows a more specific hybridization at temperatures between 55 and 65°C. This linker forms a "bubble-like structure" which itself is not involved in priming, rather it delineates the boundary between two parts. It therefore generates two recognition reactions of the primer on the target sequence. According to the manufacturer (see http://www.seegene.com/en/research/core_020.php), the 5' end (approximately 20 bases) binds preferentially to the matrix and initiates stable annealing acting as a "stabilizer". The 3' end is shorter (approximately 10 bases) and binds afterwards to the target site but only if the first step has taken place without a mismatch. The 3' end determines a target-specific extension and acts as a "determiner". Therefore, although the longer 5'-segment binds to a non-target site, the shorter segment resists non-specific extension. The short 3'-portion alone fails to make a priming at an annealing temperature. The latter also binds preferentially to the target and avoids non-specific binding. This PCR can be performed in any conventional thermocycler.

The performance of this PCR format for the detection of *H. pylori *23S rDNA mutations, involved in macrolide resistance was previously evaluated in a study published in 2007 by Woo et al., [9] with a 94.1% concordance between the DPO-based multiplex PCR and culture followed by a phenotypic susceptibility test.

In an article by Cho AR and Lee MK in Korean language, they also compared this method to culture and histology, and concluded that it could be used for the diagnosis of *H. pylori *infection and the determination of clarithromycin resistance [10]. However, they used a disk diffusion method which is not a generally accepted technique for testing *H. pylori *antimicrobial susceptibility. The present study is a retrospective study performed by the National Reference Centre for Helicobacters in France which aimed at investigating the performance of the Seeplex^® ^ClaR-*H. pylori *ACE detection kit (Seegene, Seoul, Korea) in comparison to standard phenotypic tests as well as the real-time fluorescence resonance energy transfer (FRET)-PCR developed and routinely used in our laboratory [7].

## Methods

### Materials

The Seeplex^® ^ClaR-*H. pylori *ACE detection kit was evaluated retrospectively on DNAs extracted from 127 gastric biopsies received at the French National Reference Centre for Helicobacters (Bordeaux, France) during the year 2009. There was no preselection according to the gastric site. Consecutive biopsies were included until about half of the number of biopsies positive for *H. pylori *was attained.

### Methods

The performance of the kit was compared to conventional bacterial culture followed by the determination of susceptibility to clarithromycin by E-test, and an in-house real-time PCR detection using the FRET technology [7].

### Culture

*H. pylori *strains were obtained from the corresponding gastric biopsies, after culture on Wilkins-Chalgren agar plates (Oxoid, Dardilly, France) supplemented with human blood (10% v/v) and antibiotics (10 μg/ml of vancomycin, 10 μg/ml of cefsulodin, 5 μg/ml of trimethoprim, and 10 μg/ml of amphotericin B) under microaerobic conditions, as already described [11,12]. Forty-four culture positive cases were finally included.

### Phenotypic susceptibility testing

Susceptibility to clarithromycin was assessed using the E-test method (bioMérieux, Marcy l'Etoile, France) performed as previously described [6] and using the EUCAST breakpoints: S ≤ 0.25 μg/ml; R > 0.5 μg/ml (http://www.eucast.org/clinical_breakpoints/).

### DNA extraction from gastric biopsies

Genomic DNA from gastric biopsies was extracted by using the MagnaPure LC DNA Isolation Kit I and the MagnaPure LC Isolation Station (Roche Applied Science, Penzberg, Germany). DNAs were stored at -20°C until required for analysis.

### Real-time FRET-PCR

The real-time FRET-PCR is designed to detect clarithromycin susceptible *H. pylori *(wild type) and the mutations responsible for clarithromycin resistance: A4142G and A2143G, without distinguishing between them, as well as A2142C. This test was performed as previously described [7].

### DPO-PCR

DPO-PCR was performed using the Seeplex^® ^ClaR-*H. pylori *ACE detection kit according to the manufacturer's recommendations (Seegene distributed by Eurobio Laboratoires, Courtaboeuf, France) and analyzed using a semi-automated system called Screen tape^® ^allowing an ultra rapid migration and analysis of the PCR products in small polyacrylamide gels. 8-methoxysporalen was added during the mix preparation to intercalate between double-stranded nucleic acids generated during amplification, thereby limiting carry-over contamination after UV irradiation and before PCR product analysis. The Seeplex^® ^ClaR-*H. pylori *ACE detection kit includes 3 primer pairs with a DPO structure which allows amplification of the *H. pylori *23S rDNA (621 bp amplicon) and detection of A2142G and A2143G mutations (194 bp and 475 bp, respectively). The kit also includes a primer pair for internal control.

DPO-PCR is a multiplex PCR that can be performed in any standard thermocycler.

### Histology

Briefly, histological preparations were stained with hematoxylin and eosin and Giemsa stains and the presence of *H. pylori *was evaluated according to the Sydney system. Histological results were used only in case of discrepant results obtained between DPO-PCR and FRET-PCR.

### Evaluation of Sensitivity and Specificity

The proportion of positives by DPO-PCR among the true positives defined the sensitivity and the proportion of negatives by DPO-PCR among the true negatives defined the specificity.

## Results

Concerning the 127 biopsies included in the study, culture was positive for *H. pylori *in 44 cases (34.6%), the real-time FRET-PCR in 60 cases (47.2%), and the DPO-PCR in 57 cases (44.9%). *H. pylori *status obtained from histological diagnosis was available for only 89 patients.

For the 44 biopsies positive by culture, the real-time FRET-PCR and DPO-PCR were also positive, except in one case where DPO-PCR was solely negative. For 67 biopsies, culture, FRET-PCR and DPO-PCR were all negative. Out of 16 biopsies negative by culture, FRET-PCR, DPO-PCR and histology were all positive for 14. For the remaining 2 biopsies, real-time FRET-PCR was solely positive (Tables 1 and 2) and histology confirmed the presence of *H. pylori*.

**Table 1:** Global results obtained for different diagnosis tests for the detection of *Helicobacter pylori *in human gastric biopsies

Culture (n = 44)	FRET-PCR (n = 60)	DPO-PCR (n = 57)	Total (n = 127)
(+)	(+)	(+)	**43**
(+)	(+)	(-)	**1**
(-)	(-)	(-)	**67**
(-)	(+)	(+)	**14**
(-)	(+)	(-)	**2**

The numbers indicated in parentheses represent the total number of positive samples for each test.

Using culture as the reference test, the sensitivity of DPO-PCR and real-time FRET PCR was 97.7% and 100%, respectively, and the specificity was 83.1% and 80.7%, respectively.

The concordance between real-time FRET-PCR and DPO-PCR in our study was 95% (57/60) (Table 2). Considering the 44 *H. pylori *strains isolated by culture, clarithromycin susceptibility results were available for 43 (one strain being lost after subculture). Table 2 summarizes the results obtained by the three methods used for determining macrolide susceptibility. For the 17 clarithromycin susceptible isolates, 15 corresponding biopsies contained a wild type isolate in both PCR formats. One biopsy was categorized as wild type by FRET-PCR and as an A2143G mutant by DPO-PCR. The remaining biopsy was considered to be a mixture of wild type and A2142G/A2143G mutant by FRET-PCR whereas DPO-PCR detected an A2143G mutation.

**Table 2:** Comparison of *Helicobacter pylori *susceptibility to clarithromycin by E-test, real-time FRET-PCR and DPO-PCR on human gastric biopsies

E-test	FRET-PCR	DPO-PCR	Total (n = 60)
R	R*	R^$^	25
WT	WT	WT	15
WT	R^μ^	R	1
WT	WT	R	1
R	WT	NEG	1
ND	R	R	4
ND	WT	WT	11
ND	WT	NEG	2^§^

R: macrolide resistant strain; WT: wild type (macrolide susceptible strain); NEG: negative result; ND: not determined.

* 3 biopsies with a mixture of a wild type and A2142G/A2143G mutants.

^μ ^1 biopsy with a mixture of a wild type and A2142G/A2143G mutants.

^$ ^1 double population A2142G + A2143G.

^§ ^positive histology.

For the 26 macrolide resistant isolates, a 23S rDNA mutation was detected in 25 biopsies by both PCR formats. One biopsy was considered as a wild type by real-time FRET-PCR but was negative by DPO-PCR (histology was also negative).

Globally, E-test and DPO-PCR were concordant in 95.3% of these cases (41/43).

## Discussion

We found a good correlation for the detection of *H. pylori *and the detection of clarithromycin susceptibility between the DPO-PCR and the real-time FRET-PCR routinely used in our Reference Centre. Overall, the performance is very good for a non-real-time PCR format. Compared to other PCR formats developed to detect mutations involved in macrolide resistance for *H. pylori*, DPO-PCR requires no investment in additional technical or expensive detection devices. One disadvantage is that users must run the detection of PCR fragments themselves on a 2% agarose gel before analyzing the PCR bands obtained, compared to real-time PCR formats available to date where PCR amplification is monitored automatically. DPO-PCR is therefore more time-consuming. However, in our study the semi-automated system called ScreenTape^® ^was used. ScreenTape^® ^simplifies the analysis of the results of this multiplex PCR assay.

The cost of the test is highly dependent on the activity and equipment of the laboratory in which the test is performed; however, it is significantly higher than the cost of the in-house method tested in parallel in the present study.

DPO-PCR detected more *H. pylori *positive biopsies than culture alone, with an excellent correlation with the FRET-PCR. Woo et al., identified 49 *H. pylori *positive samples among 165 culture-negative specimens using DPO-PCR [9]. This result leads us to believe that there is no specificity problem regarding DPO-PCR, rather a problem of sensitivity regarding culture.

The excellent correlation between DPO-PCR and E-test susceptibility is in line with the previous study published by Woo et al., where they found a 94.1% concordance between both methods [9]. In the work of Woo et al., two strains categorized as susceptible by E-test appeared resistant by DPO-PCR. Cho et al., also described that the results of PCR and E-test on 3 of the 8 mutation-positive biopsies were discrepant [10]. In the present study, DPO-PCR detected resistances missed by E-test also in two cases (Table 2). This could be explained by the detection limit of the Seeplex^® ^ClaR-*H. pylori *ACE detection kit which is 100 copies/reaction (100 copies/3 μl DNA). According to Woo HY et al., DPO-PCR can detect mutants present among wild-type strains at a level as low as 2% and more than 100 copies/20 μl [9]. For such a low proportion, the E-test method missed a resistant strain.

The Seeplex^® ^ClaR-*H. pylori *ACE detection kit does not allow detection of the A2142C mutation. However, this mutation is less common (usually <5% of resistant isolates) [7,5].

As indicated in the Materials and Methods, the Seeplex^® ^ClaR-*H. pylori *ACE detection kit includes 3 primer pairs with a DPO structure which allows amplification of the *H. pylori *23S rDNA (621 bp amplicon) and detection of the A2142G and A2143G mutations (194 bp and 475 bp, respectively). The first primer pair is designed to hybridize regardless of the presence of any mutation inside the PCR fragments. In the case of the A2142G mutation, its specific primer hybridizes and generates a 194 bp PCR product with the reverse *H. pylori *23S rDNA primer. In the case of the A2143G mutation, its specific primer hybridizes and forms a 475 bp PCR product with the forward *H. pylori *23S rDNA primer. Therefore, it is not possible to distinguish between 1) gastric biopsies containing a mixture of a wild-type strain and a mutated strain and 2) biopsies containing only a mutated strain (the 621 bp band corresponding to amplification of *H. pylori *23S rDNA is almost always present). It has no practical consequences because the detection of a resistant population is sufficient to exclude macrolides from the eradication therapy to be implemented.

Moreover, for 22 biopsies where A2143G was detected by DPO-PCR, the corresponding 475 bp amplicon was alone in only 4 cases which means that for these corresponding DNAs the reverse *H. pylori *23S rDNA apparently failed to hybridize and to generate the additional 621 bp amplicon. We believe that this is the reason why, in some rare cases, false negatives by DPO-PCR can occur (a total of 3 in the present study). The primer pairs which allow the amplification of the *H. pylori *23S rDNA could be slightly modified to avoid this problem.

## Conclusion

Users should keep in mind that whenever possible *H. pylori *culture should be performed, and only in cases where standard microbiology fails, the use of molecular methods are really indicated. The rationale behind this is that not only clarithromycin resistance is of interest but also that of other antimicrobials like tetracycline, quinolones, rifamycins and metronidazole. However, the Seeplex^® ^ClaR-*H. pylori *ACE detection kit is an excellent molecular test to detect *H. pylori *in gastric biopsies and to study its sensitivity to clarithromycin, especially in laboratories without expertise in culturing this bacterium and without a real-time PCR apparatus. At a time when clarithromycin resistance is increasing (prevalence is >20% in many countries), clinical laboratories could be enticed by this new PCR format.

## Competing interests

The authors declare that they have no competing interests.

## Authors' contributions

PL and FM analyzed the data and wrote the paper. ES performed the research.

All authors read and approved the final manuscript.

## Pre-publication history

The pre-publication history for this paper can be accessed here:

http://www.biomedcentral.com/1471-230X/11/112/prepub
